# Eat Slowly

**Published:** 1871-06

**Authors:** 


### EAT SLOWLY.

MANY a man has been choked to death in attempting to swallow his food before he has chewed it long enough.

Food in the stomach, surrounded with its juices, is like pieces of ice in a glass of water; for as the ice melts from without inwards, so the stomach juices dissolve the bits of food from without inwards; and, as the smaller the pieces of ice, the sooner are they melted, so the smaller the bits of food, the sooner are they dissolved, and pass out of the stomach, to be distributed to the system, giving it life, and warmth, and vigor. But if the pieces of food are large, they begin to rot before they are melted, causing heaviness, belching, nausea, or other discomforts. These make bad blood, contaminating the breath, sending dullness to the head, depression to the spirits, and a universal feeling of unwellness, lasting sometimes for half a day or a whole night. Therefore eat slowly, with deliberation; talk a great deal at meals; cultivate cheerful conversation; and let any man or woman be considered a domestic enemy and pest, who says or does anything at the table calculated to cause a single unpleasant sensation in any one present; and for the same reason have sharp knives to cut up every piece of meat as fine as a pea; and taking at least half an hour for a joyous meal, you may snap your fingers at dyspepsia and its interminable retinue of horrible symptoms.

